# “I cannot say no when a pregnant woman needs my support to get to the health centre”: involvement of community health workers in Rwanda’s maternal health

**DOI:** 10.1186/s12913-020-05405-0

**Published:** 2020-06-09

**Authors:** Germaine Tuyisenge, Celestin Hategeka, Isaac Luginaah, David F. Cechetto, Stephen Rulisa

**Affiliations:** 1 Department of Geography, Simon Fraser University, Vancouver, BC Canada; 2 Department of Geography, Western University, London, ON Canada; 3 Centre for Health Services and Policy Research, School of Population and Public Health, Faculty of Medicine, University of British Columbia, Vancouver, BC Canada; 4 Collaboration for Outcomes Research and Evaluation, Faculty of Pharmaceutical Sciences, University of British Columbia, Vancouver, BC Canada; 5 Schulich School of Medicine and Dentistry, Department of Anatomy & Cell Biology, Western University, London, ON Canada; 6 College of Medicine and Health Sciences, University of Rwanda, Kigali, Rwanda

**Keywords:** Community health, Community health workers, Maternal health services, Rwanda

## Abstract

**Background:**

In Rwanda, maternal community health workers (M-CHWs) are involved in the country’s overall health system. In maternal health, their role includes the provision of preventive and promotional health services at the community level. They provide services such as health education on maternal health wellbeing, advice and information on access and timely utilization of health facilities for prenatal, delivery and postpartum care. The contribution of M-CHWs in the health sector combined with other government initiatives led the country to achieving the fifth Millennium Development Goal (MDG) - target 5A- that aimed to improve maternal health through the reduction of maternal mortality ratio by 75% between 1990 and 2015). The objective of this study was to explore M-CHWs’ perceptions and experiences on access and provision of maternal health services.

**Methods:**

We used a case study methodology, a qualitative research approach to explore M-CHWs’ experiences and perceptions on access and provision of maternal health services at the community level in Rwanda**.** For the period of June–August 2014, in-depth interviews were conducted with sixteen M-CHWs who had been providing maternal health services in the Eastern Province of Rwanda. Participants shared their experiences and perceptions on access and provision of maternal health service in their communities.

**Results:**

The results of this research highlight the role of M-CHWs in promoting the use of health facilities for prenatal care and delivery and the ways they use to reach out to women. Several challenges prohibit M-CHWs to deliver adequate maternal health services and these are related to the poor resources settings in which they operate.

**Conclusion:**

The results of this study highlight the experiences and perceptions of M-CHWs on the provision and access to maternal health services in their communities. The fact that M-CHWs are volunteers operating in limited resources settings with no formal training in maternal health and with considerable workloads translates into challenges regarding the quality and quantity of services they provide in their communities. Such challenges create an impact on M-CHWs service provision, satisfaction and retention. The voices of M-CHWs and the communities they serve are needed to explore areas that are specific to each community context that would contribute to making the M-CHW program sustainable to achieve equitable access to maternal health services.

## Background

Involving community health workers in the health sector is among the strategies that some low and middle income countries (LMICs) have used to improve maternal health outcomes [1]. In Rwanda, community health workers in charge of maternal health have contributed to the improvement of maternal health through their participation in promotional, preventive and curative health programs in which they are involved in at the community level [2–5]. In Rwanda, maternal community health workers (M-CHWs) are volunteers selected from their villages to link communities to the formal health system. They operate as part of Rwanda’s CHW program, an integral part of the country’s health system [6]. M-CHWs’ roles involve educating the community on timely and adequate utilization of health facilities [7] and they play a direct role within their communities where they are in close contact with women who need maternal health services [2]. In addition, they provide education and information on overall health and wellbeing during pregnancy and after delivery [1].

The information they provide includes the use of antenatal services, information and services on family planning as well as information on behaviour change for better maternal health outcomes [2, 8]. As part of their responsibilities, they visit women of reproductive age in their catchment area at least once a month (the size of a catchment area per M-CHW is about 100 households) to identify those who are pregnant and encourage them to use maternal health services in a timely fashion. They also conduct mass education during community gatherings and provide a monthly report to the health centre on things like how many pregnant they have in their village, their antenatal appointment and due dates. Wherever needed, M-CHWs accompany women in labour to the health facility. Besides, M-CHWs follow up newborns for up to 1 year. They pay four mandatory visits to the newborn along the course of 1 year, and these may increase depending on the health of the baby [2, 9].

The inclusion of CHW program in the Rwandan health system combined with other initiatives in the health sector have contributed to positive maternal outcomes in the last two decades. For example, the rate of contraceptive use increased from 4 to 45.1% within a period of 10 years from 2000 to 2010 [3, 10]; the number of home deliveries reduced from 74 to 31.1% in the same period whereas the number of birth attended by skilled birth attendants doubled from 31 to 69% [4, 10]. The latest Rwanda Demographic Health Survey [11] reports that 99% of pregnant women attended to at least one antenatal care visit during their pregnancy period. It is important to also note a decrease in total fertility rate from 6.2 to 4 children from 1992 to 2013 [12]. The involvement of M-CHWs in the health system has contributed to the progress in Rwanda’s maternal health and most importantly, community members benefit greatly from the continuous maternal health education provided to them by M-CHWs [2].

Several studies have been conducted on the involvement of CHWs in different countries’ maternal health promotion efforts. A number of factors have been reported to be associated with CHWs service delivery within their communities. These include their recruitment in terms of profiles and numbers, the training, supervision and mentorship they receive, their roles and responsibilities as well as the compensation of their services [1, 13]. Other studies have focused on the relationships between CHWs and health facilities as well as their relationships with the communities they serve [13–15]. However, there is a gap in literature regarding CHWs’ own views on the services they provide in maternal health and what they believe is still needed to achieve to better maternal health outcomes. The research question we aimed to answer was “what are the perceptions and experiences of M-CHWs on maternal health service access and provision in Rwanda?”

The objective of this study was to explore M-CHWs’ experiences and perceptions the provision of maternal health services and in promoting access to maternal health services at the community level in Rwanda. This paper is one in a series of three from a master’s research that explored the experiences of access to maternal health services at the community level in Rwanda. The present paper is based on the findings from M-CHWs’ perceptions and experiences on access to maternal health services and their involvement in service provision. The first paper reports on the perceptions and experiences of women [9], whereas the second paper highlights the opinions of health professionals on access to maternal health services at the community level and their experience on service provision after receiving in-service training on maternal health care [16]. This study was funded by the Maternal, Newborn and Child Health (MNCH) project in Rwanda (2012–2015) through a Canadian International Development Agency (CIDA) grant that aimed to contribute to the promotion of maternal, newborn and child health in Rwanda. The overall study provides insights into the contributions of the MNCH project [9, 16]. It gives, through the current research, ideas for future areas of focus in terms of promoting maternal health at the community level in Rwanda and, in particular, the M-CHW program.

## Methods

### Study design

We used a case study research design to explore M-CHWs’ perceptions and experiences on access and provision of maternal health services. The case study approach was deemed appropriate for this research in order to explore the complex aspects of M-CHWs’ roles in the context of providing maternal health services [17, 18] . Through this approach, we conducted in-depth interviews with M-CHWs where they provided a deeper understanding of their experiences on access and provision of maternal health at the community level. We used a semi-structured interview guide that included open ended questions to elicit views from M-CHWs about: 1) their understanding of maternal health; 2) the types of maternal health services provided at the community level by M-CHWs; 3) the factors that contribute to the use of maternal health services; 4) gaps and barriers in the provision of maternal health services in the community; and 5) suggestions to improve service provision. All the sampled participants accepted to fully participate in the study. Body languages and non-verbal communication were observed, and field notes were taken. These were used during the analysis to better interpret the interviews.

### Data collection

The current study reports on data collected as part of a master’s research conducted in the Eastern and Western provinces of Rwanda [19]. For this study, we used data from semi-structured interviews with M-CHWs [19] in the Eastern Province, where the MNCH project operated. The project interventions included in-service training of health professionals (nurses, midwives and physicians) on maternal, newborn and child health care. The trained health professionals, especially nurses, work closely with M-CHWs. The lead author (GT) conducted interviews in Kinyarwanda, the official language in Rwanda, which is also her mother tongue. GT is familiar with the Rwandan culture as well as the Rwandan health system, which helped her to navigate the communities where the study was conducted.

The Eastern Province is the largest and most populated province with the lowest population density in the country [20]. Data were collected in eight district hospitals across the province: Nyamata, Rwamagana, Kibungo, Kirehe, Gahini, Kiziguro, Ngarama and Nyagatare. Each of these hospitals serves as a second level of health care for about 11–20 health centers. Health centers provide primary health services, and they oversee M-CHWs activities in different communities. For every health center, there are about 20–38 M-CHWs, each one of them operating in their own village of approximately 100 households and all together serving the population in the catchment area of a health center. All the M-CHWs included in this study operate in equally remote settings from the health centers.

The sample size was determined by the qualitative nature of this study. In total, sixteen semi-structured interviews were conducted with M-CHWs on their perceptions and experiences on access and provision of maternal health services in their communities. There was at least one interview per hospital selected for this particular analysis. Eligible participants were identified and were given written and verbal information about the study by the coordinators of M-CHWs’ activities at the health center and district hospital levels. The letter of information stated the objective of the study and that there was no impact whatsoever on M-CHW following their (non) participation in the study. Those who were interested in participating in the study contacted the researcher, who in return, scheduled a meeting with each of them to further explain the study, answer questions, and schedule interviews. Following participants’ choice, most interviews took place at the health center (*n* = 12), very few took place at the M-CHW’s home (*n* = 4). The semi-structured interviews were about 30–45 min and proceeded in an open dialogue form and were audio recorded with participants’ permission.

### Data analysis

All the interviews were conducted in the Kinyarwanda language and were audio-recorded with participants’ permission. The audio recordings were transcribed verbatim, translated from Kinyarwanda into English prior to analysis. Three members of the research team (GT, CH & SR) are fluent in both English and Kinyarwanda which helped for translation validity. The transcripts were stored and organized with NVIVO™ software, a qualitative data management program. Selected transcripts that corresponded to research objectives were independently reviewed by each team member. Initially, inductive coding was done by GT using NVivo and simultaneously, by each research team member manually.

A first analysis team meeting involved the discussion on codes and categorizing them into emerging themes [21]. Team members separately went back through the transcripts prior to a second meeting that was aimed to discuss on additional inductive and deductive codes for the application of thematic analysis. Thematic analysis was conducted involving the grouping of themes that emerged from the interviews in comparison to existing literature on CHWs’ involvement in promoting maternal health [4, 22–25] . Through thematic analysis, we developed different concepts and categories from the data: promoting access to maternal health services in communities, reaching out to women, and empowering M-CHWs. In order to verify the accuracy of our analysis, we returned the findings to study participants to gain their feedback and ensure that the final result and clustering were faithful and true to the data. This process helped to validate the data while offering gratitude and paying respect to acknowledge participants’ contributions.

### Ethical consideration

Prior to commencement of the study, ethical approvals to conduct this study were granted by Western University’s Research Ethics Board (File No: 103945) and Ministry of Education of Rwanda (MINEDUC/ S&T/251/2014). Before conducting interviews, letters of information were given to participants and written informed consent were obtained from them. Participants were assured of voluntary participation, confidentiality, anonymity and freedom to withdraw from the study at any time. Participants were given a token of appreciation for their time and contribution to the study. The token consisted of a food item (a bag of rice or sugar) as per the cultural protocols and participants’ socio-economic positions.

## Results

### Characteristics of the study participants

All participants (*n* = 16) were M-CHWs who provided maternal health services in their own communities. They were all females aged between 36 and 56 and were either married (*n* = 10) or divorced, separated or widowed (*n* = 6). Participants had between two and five children. Interviewees reported to have completed primary school with a small number of them (*n* = 3) who did an additional one to 3 years of high school. All participants but two reported farming as their main income-generating activity and two reported being artisans. Participants’ years of experience as M-CHW ranged from 5 months to 7 years. M-CHWs indicated that their operating zones are usually about one hundred households. The following section groups the study results into three themes that emerged from the analysis: (1) promoting access to maternal health services in communities; (2) reaching out to women and (3) empowering M-CHWs: Motivation, mentorship and training. We use direct quotes where applicable to allow participants’ voices as they express their experiences and perceptions on access and provision of maternal health services in their communities.

### Promoting access to maternal health services in communities

M-CHWs who took part in this study generally agreed on their role in the provision of maternal health care: M-CHWs frequently educate the community encouraging them to use health services available in the community such as family planning and pregnancy related services offered in health facilities. Using a community based mHealth tracking technology known as RapidSMS, M-CHWs track pregnancy and remind pregnant women about antenatal care appointments. Through this technology, M-CHWs are in contact with health facilities regarding health statuses of women in their communities. For example, they provide monthly reports to health centers about how many pregnant women and how many deliveries occurred in their villages. At times, M-CHWs also accompany women to health center for delivery or in case of pregnancy emergency.

M-CHWs reported that in most cases, women utilize their services and this is reflected in how more and more women come to them to ask for advices or information on maternal health. However, few M-CHWS also highlighted that in some instances women tend to seek their services for child health more than for maternal health issues. This was stressed by one participant: “*Even though we provide maternal health services, women mostly seek our services when they have a sick child because we provide some medicines to children under five”.* In a similar example, another participant said that: *“some women are not yet used to come to us and ask advices or seek information on issues related to maternal health … they prefer to ask fellow women, who can sometimes give them wrong information”*. Most M-CWHs highlighted that women who seek their service are mostly in farming households or within the informal sector with little knowledge about the importance of seeking health services during pregnancy.

### Reaching out to women

The participants expressed their experiences on reaching out to women for the provision of services as well as the experiences of how women access their services in the community. The fact that M-CHWs provide maternal services in their own villages where they have been selected by the community members makes it easier for them to exercise this role. All participants mentioned that they either go to women’s households or women go to M-CHWs’ homes to seek services. As highlighted by participants, both the provision and access to these services are impacted by geographical, social and economic factors.

For example, all M-CHWs indicated that their operating zones (catchment area) are usually composed of about one hundred households. However, in the context of spatial access to women’s households, the mountainous topography of some of these zones makes the access to some households very difficult. For instance, the households located on the top of mountains or those in villages with very bad roads during rainy seasons are sometimes not easily accessible. A participant explained that “*The rainy period can be very challenging when I have to get to women’s households … some of them live in quite a distance from my house … but I cannot say no when a woman calls me and needs me to go see her.*”. In the same context, another M-CHW mentioned that: “*For example when a woman is in labor and has none to accompany her to the health center … even at night I have to go … when the road is very bad it takes me longer to walk to her place*”. All of the participants highlighted that the mode of transportation used to reach to women is walking. However, the physical access can be impacted by many factors including the distance between the M-CHW’s house and where the woman lives, the time of the day and weather conditions.

Additionally, since M-CHWs work as volunteers in their community, they are exposed to financial constraints that may impact their service provision. On the one hand, financial factors are related to the loss of income while performing M-CHWs’ duties, for example when providing maternal health services during working hours. Most participant expressed to have encountered such financial constraints, especially due to the nature of their job, since women might need their help at any given time of the day and they have to be available to provide needed support: “*even the time I spend going around households can be converted into loss income because I can use that time farming or selling produce …*. *but I cannot think like that because I’m passionate about improving my community*”. A few M-CHWs also face financial challenges when dealing with poor households, for example, when a woman in labor needs to reach to a health facility but does not have health insurance or baby items. In such cases, a few M-CHWs reported that they happen to take care of such expenses using their own money with hope that they will be reimbursed by the women, but in most cases, this does not happen.

Similarly, few M-CHWs happen to use their own financial resources to pay for transportation to get women to the health centre. In this context, a participant said: *“I happen to spend my own money for transportation, communication or for any other unpredictable expenses... it’s the nature of our job … Of course, it would be nice to have compensation or at least get reimbursed”*. M-CHWs do not receive any travel allowance or reimbursements for any money spent paying costs for their clients; however, participants highlighted that these are losses that they expect due to the nature of their job of helping pregnant women. Finally, M-CHWs reported about other factors that impact their service provision. For example, a few participants highlighted that their acceptability depends on the understanding of community members and their family members about maternal health and the roles of a M-CHW. In this context, one participant expressed that: “*some women refuse our services … especially teen girls or women with many children tend to hide their pregnancies … even when we go to them they may deny being pregnant or may not receive us in their homes … it’s hard to follow them up*”. A few participants stressed that these constraints can impact negatively their service delivery and overall maternal outcomes in their villages.

### Empowering M-CHWs: motivation, mentorship and training

Participants highlighted that due to the volunteering nature of their job, they have to do other income generating activities during the day and volunteer as M-CHWs in the evenings, which is not always easy. One M-CHW commented that: “we *have many tasks as M-CHWs and we have to submit a daily report of our activities … we do our best to improve community health but unfortunately we have to do other income generating activities for our families”*. Most of M-CHWs also expressed that they do not have enough training to accomplish their roles in providing maternal care and sometimes they are not able to respond to all the concerns that women may have.

Despite these challenges, all the participants said that there has been remarkable increase in utilization of maternal health services. All of the M-CHWs pointed out that they receive training from health centers; hence they are getting empowered in their activities, which they believe has contributed to the increase of community trust in M-CHWs services. Training is usually organized by the Ministry of Health or by other stakeholder organizations in maternal health. In this regard, an interviewee highlighted the following: *“we get different training to increase our knowledge and skills in maternal health. This has increased our confidence, when for example we are asked questions about family planning methods. Now we can answer to the questions or give advices to women”*. M-CHW training has involved how to follow-up pregnant women until delivery and after including education on physical activity and nutrition issues during pregnancy, signs and symptoms that may require emergent/urgent care and care during postpartum period such as breastfeeding and family planning services. M-CHWs mentioned that there is irregularity in receiving training and a lack of ongoing mentorship for their activities.

## Discussion

The current study provides perceptions and experiences of M-CHWs on access and provision of maternal health services to promote timely utilization of these services and highlights different ways through which M-CHWs achieve this. The Rwandan health system is structured with a community focus and in maternal health, M-CHWs are the first point of contact. M-CHW services are especially important in remote communities where they serve as the primary link to the health system [26]. A study conducted in Bugesera, Rwanda [4] on the prevalence of giving birth in a health facility, reports that 43% of 859 women who gave birth within 3 years received at least one visit by an M-CHW during the life course of their pregnancy. Moreover, 95% of these women stated that M-CHWs advised them to deliver in health facilities In this study, M-CHWs reported that in addition to making regular home visits, they usually accompany the woman to the facility for delivery and whenever there is a problem. As stressed by this study, M-CHWs encounter instances that require them to be resourceful in ways that sometimes go beyond their capabilities. For example, M-CHW’s often report that they use their own limited resources to pay for pregnant women’s transportation. Because these unpaid volunteers receive no salary or reimbursement for expenses, these costs are an economic burden for them and their families Different studies conducted in LIMCs have highlighted the role of CHWs in improving maternal health [6, 22, 24] . Surprisingly, even though different countries have incorporated CHWs in their maternal health systems, in most of these countries it is still unclear what exactly CHWs roles are and where their responsibilities start and end [13]. In addition, CHWs roles and responsibilities in maternal health are often unspecified and the responsibilities of governments towards CHWs, including their recruitment, training, retention and their compensation are uncertain [13].

M-CHWs’ roles differ in different health systems and communities, depending in part on available resources [20, 27] . Similarly to any health worker involved in maternal health service delivery, M-CHWs act with urgency in most situations to save both the mother and the baby. Hence the burden to fulfill this role could be high on M-CHWs, mainly when operating in limited resources settings [6, 28]. There is a need of programs targeting the promotion of capacity building and community based empowerment of M-CHWs that would impact the access and provision of maternal health services and overall outcome of maternal health in Rwanda.

## Conclusion

The results of this study highlight the experiences and perceptions of M-CHWs on the provision and access to maternal health services in their communities. The fact that M-CHWs are volunteers operating in limited resources settings with no formal training in maternal health and with considerable workloads translates into challenges regarding the quality and quantity of services they provide in their communities [1, 27]. Such challenges create an impact on M-CHWs service provision, satisfaction and retention. The voices of M-CHWs and the communities they serve are needed to explore areas that are specific to each community context that would contribute to making the M-CHW program sustainable to achieve equitable access to maternal health services.

## Data Availability

The datasets generated and analysed during the current study are not publicly available due to the respondents’ consent to use the data for this research specifically. Data can be available upon reasonable request to the corresponding author.

## Abbreviations

CHW: Community Health Worker
M-CHW: Maternal Community Health Worker
FCHV: Female Community Health Volunteer
LMIC: Low- and Middle-Income Country
MDG: Millennium Development Goal
